# A Golgi-Located Transmembrane Nine Protein Gene TMN11 Functions in Manganese/Cadmium Homeostasis and Regulates Growth and Seed Development in Rice

**DOI:** 10.3390/ijms232415883

**Published:** 2022-12-14

**Authors:** He Li, Chao Li, Xuesong Liu, Zhimin Yang

**Affiliations:** 1 Department of Biochemistry and Molecular Biology, College of Life Sciences, Nanjing Agricultural University, Nanjing 210095, China; 2 Institute of Agricultural Facilities and Equipment, Jiangsu Academy of Agricultural Sciences, Nanjing 210014, China

**Keywords:** OsTMN11, Golgi, Mn transporter, rice, cadmium, seed development

## Abstract

Metal transporters play crucial roles in plant nutrition, development, and metal homeostasis. To date, several multi-proteins have been identified for metal transport across the plasma membrane and tonoplast. Nevertheless, Golgi endomembrane metal carriers and their mechanisms are less documented. In this study, we identified a new transmembrane nine (TMN) family gene, TMN11, which encodes a Mn transport protein that was localized to the *cis*-Golgi endomembrane in rice. OsTMN11 contains a typically conserved long luminal N-terminal domain and nine transmembrane domains. OsTMN11 was ubiquitously expressed over the lifespan of rice and strongly upregulated in young rice under excess Mn(II)/Cd(II) stress. Ectopic expression of OsTMN11 in an Mn-sensitive *pmr1* mutant (PMR1 is a Golgi-resident Mn exporter) yeast (*Saccharomyces cerevisiae*) restored the defective phenotype and transported excess Mn out of the cells. As ScPMR1 mediates cellular Mn efflux via a vesicle-secretory pathway, the results suggest that OsTMN11 functions in a similar manner. OsTMN11 knockdown (by RNAi) compromised the growth of young rice, manifested as shorter plant height, reduced biomass, and chlorosis under excessive Mn and Cd conditions. Two lifelong field trials with rice cropped in either normal Mn supply conditions or in Cd-contaminated farmland demonstrated that knockdown of OsTMN11 impaired the capacity of seed development (including panicle, spikelet fertility, seed length, grain weight, etc.). The mature RNAi plants contained less Mn but accumulated Cd in grains and rice straw, confirming that OsTMN11 plays a fundamental role in metal homeostasis associated with rice growth and development even under normal Mn supply conditions.

## 1. Introduction

Manganese (Mn) is an essential mineral element required for plant growth and development. It is actively involved in the electron transport chain of photosynthetic system II (PSII) and enzyme activation as a cofactor [1]. Even though it is necessary, the concentration of Mn must be within a narrow window (~20–40 mg/kg of dry weight) for most plant species [2]. Manganese deficiency in plants causes severe malnutrition symptoms, such as browning and necrotic spots on leaves, and impaired reproductivity of crops [3]. In contrast, when plants grow in an excessive Mn environment, they suffer from Mn toxicity and display lower photosynthetic efficiency and impaired photosynthetic pigment content [1]. Different from Mn, cadmium (Cd) is a non-essential element that exists in natural soils, but the rising accumulation of Cd in soil due to human activities manifests itself as a kind of environmental hazmat that poses health risks to crop production and food security [4]. Rice (*Oryza sativa*) is a unique food crop that thrives in wetlands where the waterlogged soils under low pH and redox conditions usually generate massive amounts of soluble Mn (II) and Cd(II) ions that are readily absorbed by plants [5]. While excessive Mn in rice dampens metal homeostasis and many other biological processes, Cd overload in rice also blocks plant growth, reduces food quality, and causes multi-chronic diseases through the food chain [4]. Mining genetic resources related to Mn and Cd acquisition to better understand how rice plants deal with metal toxicity is needed to not only answer a fundamental scientific question, but it is needed to build up genetically improved varieties that can adapt to stress environments [6,7]. Over the last decades, a growing number of metal transporters relevant to Mn and/or Cd acquisition, translocation, and homeostasis, with potential applications in phytoremediation, have been identified in plants [8,9,10]. However, most studies have investigated metal transporters localized on the plasma membrane and tonoplast, while few metal transporters have been identified on other endomembrane networks.

Transmembrane nine (TMN or TM9) family proteins are a unique class of endomembrane proteins that are highly conserved in eukaryotic organisms such as Dictyostelium (*Dictyostelium discoideum*) [11], Drosophila [12], yeast (*Saccharomyces cerevisiae*) [13], humans [14], and plants [15]. A typical TMN protein is composed of a long luminal N-terminal domain, nine transmembrane domains, and a short C-terminal tail [16]. In animals, many studies have focused on the biological functions of TMNs, such as cell migration, vesicular transport, autophagy, Golgi vesicular trafficking, and ion homeostasis [17,18]. For example, loss of Phg1A function in Dictyostelium results in cell adhesion defects and inefficient phagocytosis [11,12,13]. In *S. cerevisiae*, Hegelund et al. [15] reported that knocking out TMN1/2/3 (triple mutant *tmn1-3*) led to a drastic reduction in copper (Cu) concentration (25%) compared to that in the wild-type, whereas transgenic yeast expressing TMN1-3 (a homologue of yeast TMN1/2/3) accumulated double the cellular Cu concentration. Furthermore, mutation of *AtTMN1* altered the synthesis of pectic polysaccharides and blocked root elongation depending on the borate supply conditions in Arabidopsis, suggesting that AtTMN1 is required for the crosslinking of pectic polysaccharides during root growth [19]. To date, 12 and 17 TMNs have been identified in Arabidopsis and rice genomes [20], respectively, but only AtTMN1 has been functionally characterized in plants.

In this study, we functionally identified an uncharacterized rice TMN family member, OsTMN11, which was previously isolated from our metal stress response libraries [21]. OsTMN11 resides in the Golgi apparatus and is dominantly expressed in rice roots. OsTMN11 expression was transcriptionally upregulated under excessive Mn and Cd stress. Ectopic expression of OsTMN11 in mutant yeast strains showed Mn and Cd transport activities but less accumulation in cells. Knocking down OsTMN11 (by RNAi) compromised the growth of young rice under excess Mn conditions and the seed development of mature rice under normal Mn supply conditions. The impaired function of OsTMN11 in RNAi plants was also connected to the decreased vegetative growth and seed development of rice grown in Cd-contaminated soil. These results suggest that OsTMN11 is required for Mn or Cd homeostasis in rice, which has implications for the application of plant RNAi for the phytoremediation of Cd-polluted wetland soils.

## 2. Results

### 2.1. Structure and Sequence of OsTMN11 Gene and Protein

**Figure 1:**
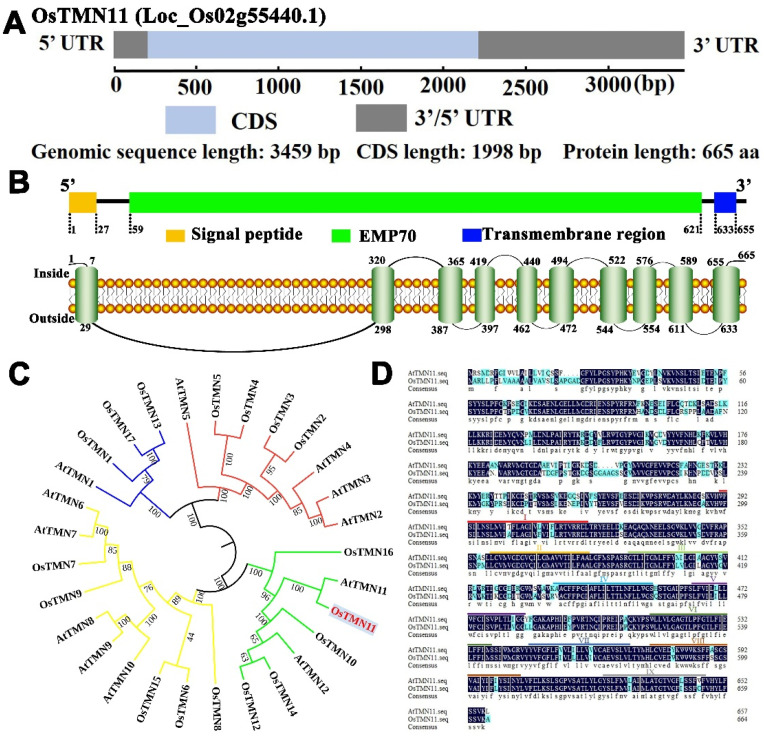
Analysis of OsTMN11 gene and protein structures and relationship with its homologues in Arabidopsis. (**A**) Diagram of *OsTMN11* structure. CDS, coding sequence. UTR, untranslated regions. aa, amino acid. (**B**) Diagram of structural transmembrane domain of OsTMN11. EMP70, endomembrane protein 70. (**C**) Phylogenetic relationship between OsTMN11 and its homologues in rice and Arabidopsis. (**D**) Diagram of sequence similarity for OsTMN11 and AtTMN11.

*OsTMN11* resides in chromosome 2 (Figure S1). The full length of *OsTMN11* is 3459 bp, including an exon of 1998 bp that encodes a protein with 665 amino acids (Figure 1A). The protein structure analysis revealed that OsTMN11 contains the typical domains of a transmembrane nine (TMD) protein and an N-terminal signal peptide. Among them, eight transmembrane helices constitute the conserved functional domain, named endomembrane protein70 (EMP70), localized within amino acids 59–621 (Figure 1B). The phylogenetic tree for all TMN proteins in rice was constructed using MEGA6.0 software, showing that OsTMN11 is closest to AtTMN11, with a sequence identity of 76.73% (Figure 1C,D).

### 2.2. OsTMN11 Is Localized to the Cis-Golgi Apparatus and Induced under Mn and Cd Stress

**Figure 2:**
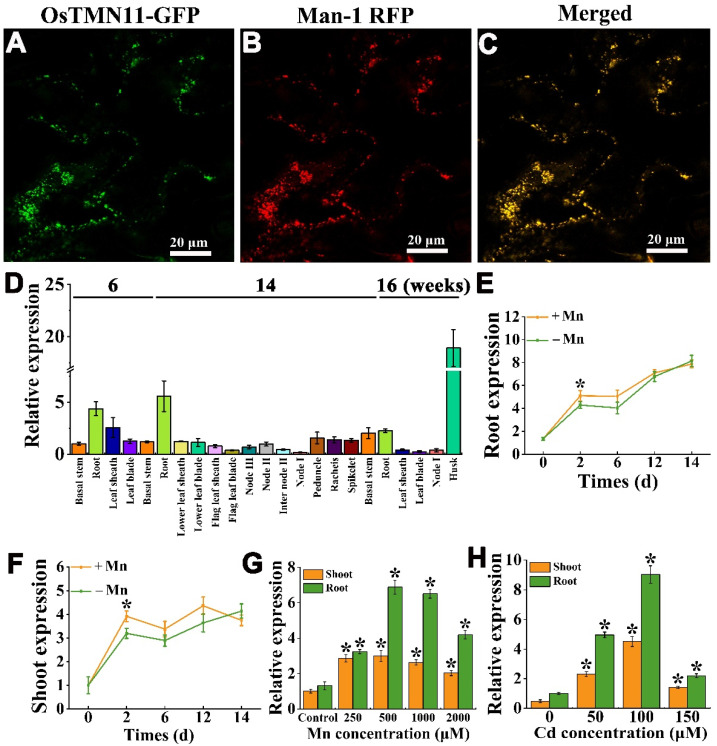
OsTMN11 subcellular localization and transcriptional expression patterns under normal conditions and Mn or Cd stress. (**A**–**C**) Subcellular localization of OsTMN11-GFP fusion protein in tobacco leaf epidermal cells detected by confocal laser scanning microscopy. (**A**) The GFP fluorescence signal of OsTMN11-GFP fusion protein only. (**B**) The red fluorescence of Golgi maker (Man-1). (**C**) The merged image of the OsTMN11-GFP fusion protein signal and the Golgi maker (Man-1 RFP for *cis*-Golgi). Bars = 20 µm. (**D**) Transcriptional expression of *OsTMN11* in different tissues or organs of rice grown at the vegetive, flowering, and developmental stages. (**E**–**G**) Two week-old rice seedlings were exposed to excessive Mn for three hours (**E**), deficient Mn for two weeks (**F**,**G**), or excessive Cd for three hours (**H**). Error bars indicate the means ± standard deviations of three biological replicates. Asterisks indicate that the mean values are significantly different between the treatment and control (*p* < 0.05).

The OsTMN11 sequence was separated and amplified using RT-PCR. The C-terminal or N-terminal of OsTMN11 was liganded to the green fluorescent protein (GFP) of the pAN580 vector at the Sel1/Kpn1 by restriction endonucleases, leading to the generation of the OsTMN11-GFP fragment driven by the cauliflower mosaic virus promoter (35S). The constructed 35S-OsTMN11-GFP vector and several kinds of intimal markers were co-expressed in the epidermal cells of three week-old young tobacco leaves through *Agrobacterium tumefaciens* transformation [22]. Our analysis showed that only the *cis*-Golgi marker Man-1 overlapped with the green OsTMN11-GFP fluorescence, which was merged into yellow punctate signals (Figure 2A–C).

OsTMN11 was transcriptionally expressed in nearly all growth and developmental stages and abundantly expressed in the roots and grains (Figure 2D). To determine whether *OsTMN11* transcription was affected by heavy metals, 14 day-old rice plants were stressed with Mn deficiency for 14 days or supplied with excess Mn for 6 h. Deprivation of Mn in the growth medium did not have much effect on *OsTMN11* expression, except at the two day time point where slight but significant changes were observed (Figure 2E,F). However, under excess Mn exposure, OsTMN11 expression was strongly induced (Figure 2G). The high expression of OsTMN11 in rice roots and shoots was observed at 500–1000 μM Mn. Evaluation of OsTMN11 transcripts under Cd stress also revealed a high induction pattern in the range of 0–100 μM Cd (Figure 2H). A similar result was detected in rice exposed to excess Zn and Fe, but OsTMN11 expression in the roots was repressed under a higher supply of Fe (Figure S2). These results indicated that transcription of *OsTMN11* can be induced by high concentrations of metals but not by Mn deficiency.

### 2.3. OsTMN11 Knockdown RNAi Lines Are Sensitive to Excessive Mn and Cd Ions

**Figure 3:**
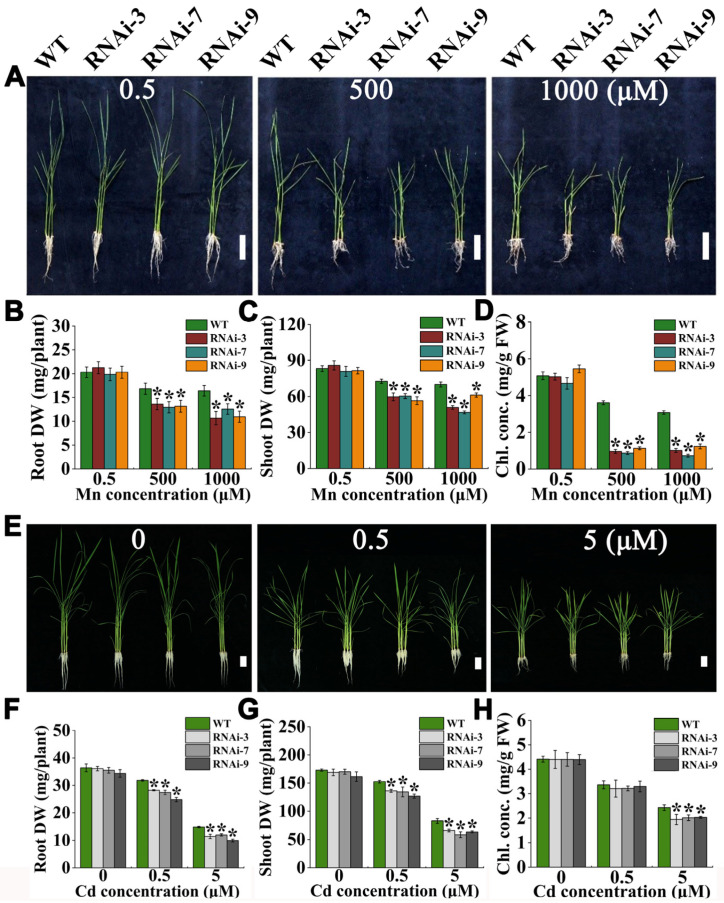
Growth responses of WT and RNAi lines to excessive Mn and Cd stress. Two week-old young rice plants were grown in nutrient solution supplemented with Mn (0.5, 500, and 1000 μM) and Cd (0, 0.5, and 5 μM) for 14 days. (**A**–**D**) Mn stress. (**E**–**H**) Cd stress. (**A**,**E**) Phenotypes of WT and RNAi lines. Bars = 5 cm. Comparative growth responses between WT and RNAi lines under Mn and Cd stress, as presented by root dry weight (DW) (**B**,**F**), shoot DW (**C**,**G**), total chlorophyll concentration (**D**,**H**). FW, fresh weight. Error bars indicate the means ± standard deviations of three biological replicates. Asterisks indicate that the mean values of three replicates are significantly different between WT and RNAi lines (*p* < 0.05).

Using the rice genetic transformation mediated by *Agrobacterium tumefaciens*, we built up a set of rice OsTMN11 RNAi lines, three of which were randomly selected for functional analysis. OsTMN11 transcript levels of three RNAi lines were measured. The OsTMN11 transcript levels of RNAi-3, RNAi-7, and RNAi-9 were downregulated to 65.2%, 30.1%, and 48.9% of the levels in wild-type rice, respectively (Figure S3). Fourteen day-old RNAi and wild-type plants were treated with 0.5 (control), 500, and 1000 μM Mn for 14 days. The RNAi lines developed a similar phenotype as the wild-type rice under normal Mn supply conditions (Figure 3A and Figure S4A,B). However, at high Mn concentrations, the RNAi lines displayed supersensitive growth phenotypes, including plant dwarfing, leaf yellowing, and loss of biomass relative to that of wild-type rice. Under 500 μM Mn stress, the plant heights of the three RNAi lines decreased by 24–37% and the dry weights declined by 16–22%(Figure 3B,C). Further analyses revealed that the RNAi plants had lower levels of chlorophyll than wild-type rice, with the chlorophyll concentrations decreased by 69–76% and 60–77% at 500 and 1000 μM Mn, respectively (Figure 3D). The phenotypes of root and shoot elongation and biomass were measured in rice under Mn-deficiency stress for 30 days. As shown in Figure S5, Mn starvation hardly affected the growth of wild-type and RNAi plants, except the shoot elongation of the RNAi lines was slightly but significantly affected compared to that of the wild-type rice. Exposure to Cd (0, 0.5, and 5 μM) also significantly reduced the growth of RNAi lines (Figure 3E). Under 5 μM Cd stress, values for the plant height, root elongation, tissue dry weight, and chlorophyll concentration of the RNAi plants declined by 16–24.7%, 14.7–19.8%, 19.5–33.2%, 20.1–29.3%, and 16.3–19.8%, respectively (Figure 3F–H and Figure S4C,D).

### 2.4. OsTMN11 Knockdown Results in Overaccumulation of Mn and Cd in Rice Plants

**Figure 4:**
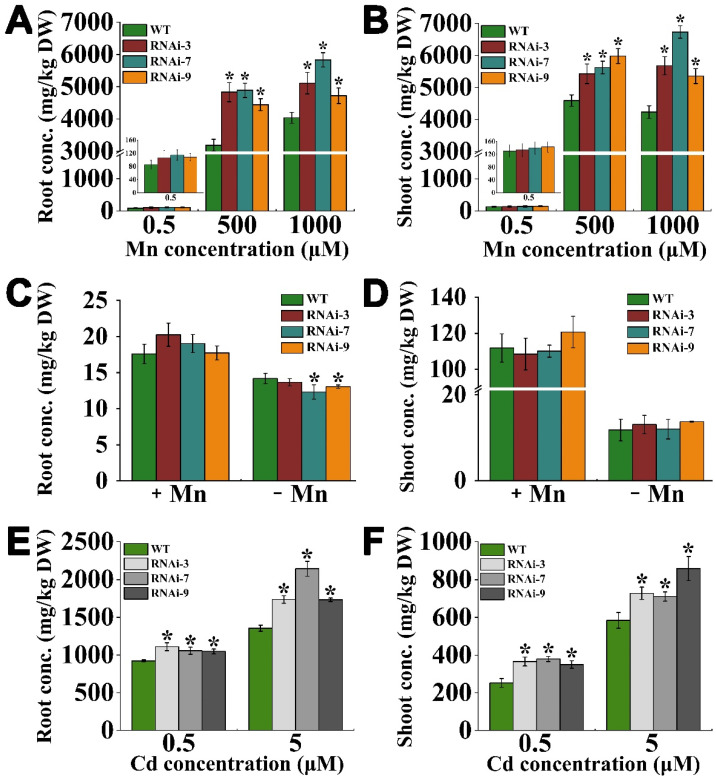
Accumulation of Mn and Cd in tRNAi and wild-type (WT) rice under Mn and Cd stress. Two week-old young rice plants were hydroponically grown with 0.5 (normal), 500, and 1000 μM Mn and 0, 0.5, and 5 μM Cd for 14 d, or under Mn deficiency for 30 d. (**A**,**B**) Mn concentrations in roots and shoots exposed to excessive Mn supply conditions. (**C**,**D**) Mn concentrations in roots and shoot under Mn deficiency (+Mn: 0.5 μM). (**E**,**F**) Cd concentrations in roots and shoots. Error bars indicate the means ± standard deviations of independent biological replicates. Asterisks indicate that the mean values of three replicates are significantly different between WT and RNAi lines (*p *< 0.05).

To investigate Mn or Cd accumulation in the RNAi lines, two week-old rice seedlings were hydroponically grown in nutrient solutions with excess Mn or Cd for 14 days. Under normal conditions, the RNAi plants accumulated slightly but not significantly more Mn in the roots and shoots, whereas high Mn supply resulted in significant accumulation of Mn in the tissues of the RNAi lines (Figure 4A,B). Under 500 μM Mn stress, the Mn concentrations in the RNAi roots and shoots were increased by 41–53% and 18–29% compared with the wild-type rice, respectively. However, deprivation of Mn from the growth medium for 30 days hardly impacted Mn accumulation in the RNAi lines, except in the roots of the RNAi-7 and RNAi-9 lines, which showed slightly but significantly lower levels of Mn (Figure 4C,D). Similarly, treatment with Cd led to a higher accumulation of Cd in the root and shoot tissues of the RNAi plants compared to that in the wild-type rice (Figure 4E,F). These results indicated that knocking down OsTMN11 would impair metal homeostasis, implying that OsTMN11 is required for proper Mn transport or accumulation in rice.

### 2.5. OsTMN11 Can Complement a Mn-Sensitive Phenotype of Golgi-Resident pmr1 Mutant Yeast and Regulate Cellular Mn Efflux

**Figure 5:**
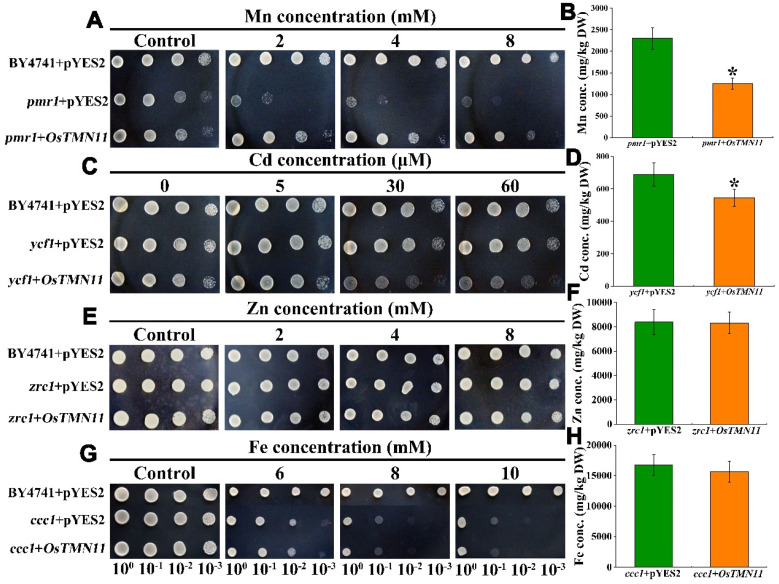
Measurement of transport activity of Mn, Cd, Zn, and Fe in *OsTMN11*-transformed yeast cells. Wild-type strain (BY4741) and sensitive mutants (*pmr1*, *ycf1*, *zrc1* and *ccc1*) transformed with *OsTMN11 *and pYES2 empty vectors and were grown in YNB solid (**A**,**C**,**E**,**G**) and liquid SD-Ura (**B**,**D**,**F**,**H**) medium supplemented with Mn, Cd, and Zn, respectively. All treated yeast cells were digested. The metal concentrations in the cells were quantified using inductively coupled plasma mass spectrometry. Error bars indicate the means ± standard deviations of three biological replicates. Asterisks indicate that the mean values are significantly different between the treatment and control or between the empty and transformed cells (*p* < 0.05).

To figure out the mechanism of OsTMN11 involvement in Mn or Cd transport, *OsTMN11* was transformed into yeast (*Saccharomyces cerevisiae*, wild-type BY4741) and its *pmr1* mutant line, which is sensitive to excess Mn. PMR1 is a Golgi-localized Mn efflux transporter working on output of subcellular Mn in the vesicular secretory pathway [23]. Deletion of PMR1 in the *pmr1* mutant results in a Mn-hypersensitive phenotype with severely blocked cellular growth and an overaccumulation of Mn ions [24]. This model is widely used for testing Mn transport, detoxification, and accumulation [7,25,26]. Our studies showed that under excessive Mn supply conditions, the growth of mutant cells carrying the null gene (*pmr1* + pYES2) was significantly inhibited compared to that of the wild-type (BY4741 + pYES2) yeast (Figure 5A). When *OsTMN11* was transformed into *pmr1* (*pmr1* + OsTMN11), growth inhibition was largely restored. Assessment of Mn concentrations revealed that the *OsTMN11*-carrying *pmrl* strain accumulated less Mn (Figure 5B). This result indicated that ectopic expression of OsTMN11 in yeast can complement the defects of the manganese sensitive phenotype. The ability of OsTMN11 to complement the *pmr1* phenotype raised the possibility that OsTMN11 might detoxify Mn by a mechanism very similar to that of PMR1 through the loading of secretory vesicles and subsequent exocytosis.

We further examined the ability of OsTMN11 to transport Cd, Zn, and Fe by transforming *OsTMN11* into yeast mutants (*ycf1*, *zrc1*, and *ccc1*) sensitive to the corresponding excess metals. YCF1 (yeast cadmium factor 1) is a member of the ABCB (ATP-binding cassette B) transporter family and is localized to the tonoplast in yeast. The knockout mutant *ycf1* displays a Cd sensitive phenotype [27]. The transgenic cells expressing OsTMN11 appeared slightly sensitive compared to the control cells (Figure 5C), but the Cd concentration in the transformants was moderately lower than that in the empty cells (Figure 5D), suggesting that OsTMN11 was able to transport Cd. With regards to Zn and Fe stress, the growth and metal concentrations of the transformed and wild-type cells did not differ significantly (Figure 5E–H).

### 2.6. OsTMN11 Suppression Dampens Rice Growth and Development under Natural Conditions

**Figure 6:**
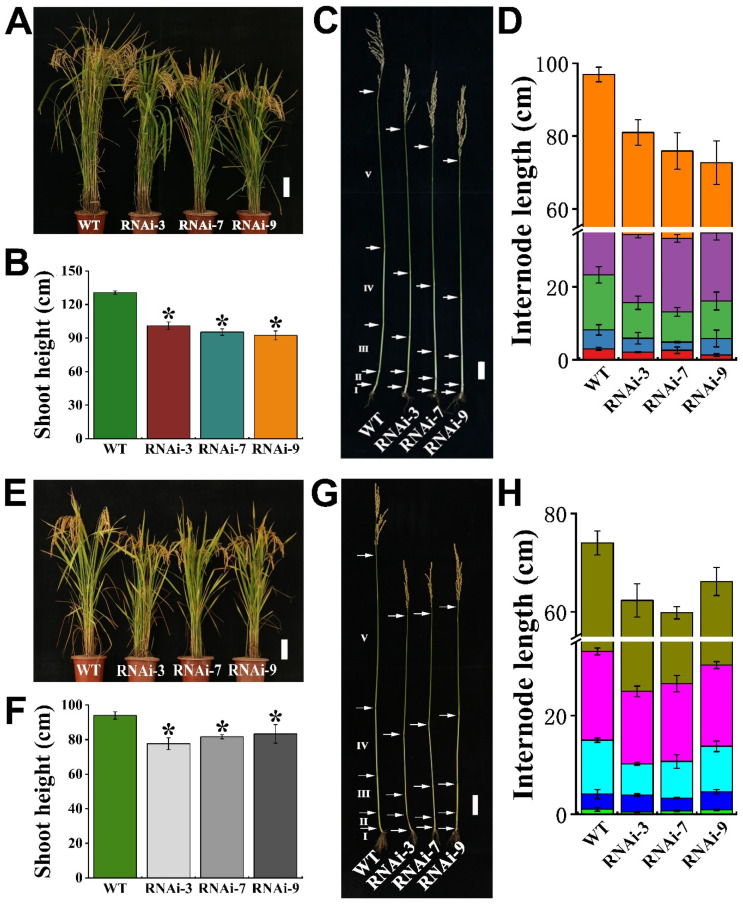
Variations in rice morphology and internode length of mature tissues between RNAi lines and wild-type (WT) rice grown on soil with and without Cd (for Mn trial) contamination. The rice varieties were ripened under natural conditions throughout the lifespan. (**A**–**D**) Rice was grown on soil without Cd and harvested to measure Mn concentrations. (**E**–**H**) Rice was grown in Cd-contaminated soil and sampled to measure Cd concentrations. (**A**,**E**) The growth phenotypes of WT and RNAi rice (Bars = 10 cm). (**B**,**F**) Shoot height of WT and RNAi lines. (**C**,**G**) Morphological culm (internode) structures of WT and RNAi (Bar = 5 cm). The arrow indicates the node position. (**D**,**H**) Quantitative analysis of internode elongation. Error bars indicate the means ± standard deviations of three independent biological replicates. Asterisks indicate that the mean values of three replicates are significantly different between WT and RNAi lines (*p *< 0.05).

To validate the short-term hydroponic study, we separately conducted two lifelong field trials on the rice varieties grown under natural conditions. Two types of soil were utilized for the studies: soil with and without Cd contamination. Local, normal, agronomic management practices for rice cultivation were adopted during the field experiments. The mature rice was harvested and the plant phenotypes were analyzed. It was found that the shoot lengths of the RNAi lines were significantly lower than those of wild-type rice, which were reduced by 22.7–29.4% (Figure 6A,B). The culm lengths of the RNAi lines were also markedly decreased compared to those of the wild-type control (Figure 6C,D). With regards to the RNAi plants grown in Cd-contaminated soil, a similar attenuated phenotype was found (Figure 6E–H).

**Figure 7:**
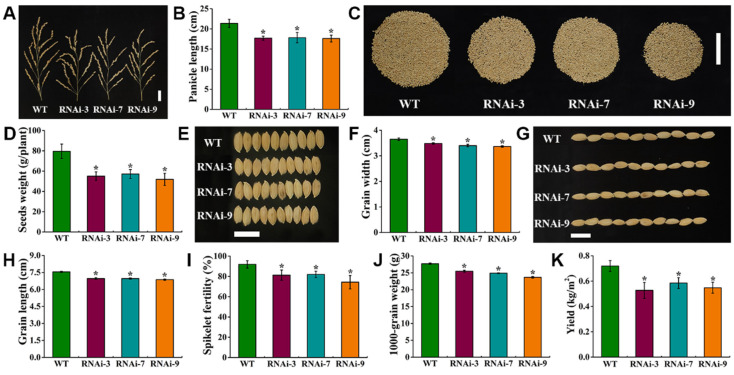
*OsTMN11* RNAi lines inhibited panicle elongation and seed development of rice, resulting in decreased rice yield. Rice was grown to maturity under natural conditions for two consecutive years, with the statistics for the second year shown. (**A**) Phenotypes of panicles (Bars = 3 cm). (**B**) Panicle length. (**C**) Phenotype analysis of yield per plant (Bar = 10 cm). (**D**) Seed weight per plant. (**E**,**G**) Phenotypes of ten grains (Bar = 1 cm). (**F**) The ten-grain width. (**H**) The ten-grain length. (**I**) Spikelet fertility per panicle. (**J**) 1000-grain weight. (**K**) Seed yield per square meter. Data represent the means of three biological replicates. The data were tested by Tukey’s test, and the asterisk indicated that there was a significant difference between WT and RNAi lines (*p* < 0.05).

We further evaluated the impact of *OsTMN11* suppression on rice seed development and reproductivity. The panicle lengths of the RNAi lines were significantly lower than those of the wild-type rice, which were reduced by 18.3–24.7% (Figure 7A,B). The seed yields of RNAi plants were significantly reduced by 28.15–34.82% (Figure 7C,D). Three groups of ten seeds were randomly selected from different lines, revealing that the ten-grain widths and lengths of the RNAi plants were reduced by 4.57–7.76% (Figure 7E,F) and 7.81–9.06% (Figure 7G,H), respectively. In addition, spikelet fertility decreased by 9.89–17.52% and the 1000-grain weights decreased by 8.05–14.51% (Figure 7I,J). Eventually, the grain yields per unit square meter were reduced by 18.78–26.70% (Figure 7K). These results indicated that knocking down OsTMN11 would jeopardize panicle development and grain plumpness, which was attributed to impaired Mn homeostasis.

### 2.7. OsTMN11 Disruption Impaired Mn and Cd Accumulation in Maturity Rice Plants

**Figure 8:**
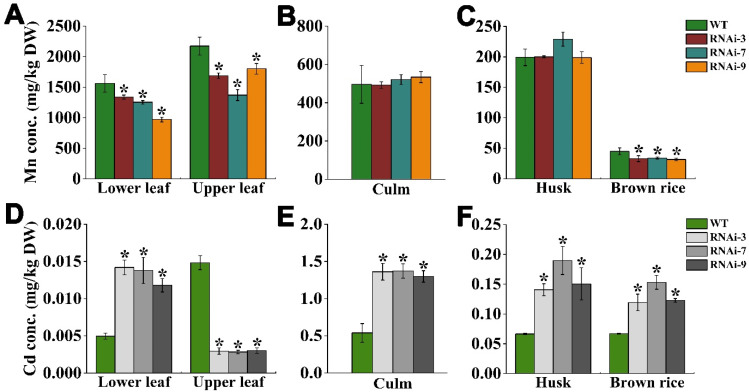
Mn and Cd concentrations in the tissues/organs of RNAi and wild-type (WT) plants at the mature stage. (**A**–**C**) Rice plants were grown in normal soil (without Cd contamination) and (**D**–**F**) in Cd-contaminated soil throughout the lifespan. When rice was ripe, total Mn and Cd concentrations in the lower leaves (three basal leaves), upper leaves (three top leaves) including leaf blade and sheath, culm, and grains were measured. Bars indicate the means ± standard deviations of at least three independent biological replicates. Asterisks indicate that the mean values of three replicates are significantly different between WT and RNAi plants (*p *< 0.05).

To explore whether OsTMN11 is able to regulate the transport and accumulation of Mn and Cd in rice, the major tissues (or organs) of the mature rice plants were separately harvested and Mn/Cd concentrations were determined by ICP-MS. The Mn concentrations in the lower and upper leaves of RNAi plants were drastically reduced compared to those in the wild-type rice (Figure 8A,B). There were no marked differences in Mn concentrations in the culm and husk between the RNAi and wild-type plants (Figure 8B,C). In contrast, the brown rice of RNAi lines contained less Mn (reduced by 24.14–29.84%) than that of the wild-type control (Figure 8C).

We also examined morphological variations in the tissues and organs related to seed development between the WT and RNAi plants grown in Cd-contaminated field soil. We found similar results for the RNAi lines, with weaker growth and developmental fitness under Cd stress (Figure S6). However, no changes were observed for the seed phenotypes of the RNAi lines compared to those of the WT rice (Figure S7). As for the RNAi lines grown in Cd-contaminated soil, the most obvious characteristic was that massive Cd accumulation was detected in the lower leaves, while Cd accumulation in the upper leaves was remarkably lowered (Figure 8D). Similar to the basal leaves, the concentrations of Cd in the culm, husk, and brown rice were found to be significantly higher (Figure 8E,F). These results suggested that disruption of *OsTMN11* resulted in abnormal Mn and Cd translocation or accumulation in rice plants.

## 3. Discussion

Manganese homeostasis is critically important for healthy crop production. Most rice genotypes or cultivars are endowed with adaptative mechanisms to deal with the changing Mn environment. One of these mechanisms concerns Mn transporters that either move Mn out of cells across the plasma membrane or sequester it into vacuoles for detoxification [28]. However, the complex Mn transport networks in the Golgi subcellular endomembrane, as part of the secretory pathway, remain elusive [9]. This study characterized a novel function of the Golgi-resident TMN11, which is required for Mn homeostasis in rice. OsTMN11 was shown to reside in the Golgi apparatus, which is similar to the recent report on TMN1 in Arabidopsis, although their biological functions are apparently different [19]. Heterologous expression in yeast revealed that OsTMN11 is involved in Mn and Cd transport. Knockdown of OsTMN11 resulted in high metal accumulation in young rice exposed to excess Mn and Cd, but did cause a response to normal or short-term Mn deficiency. The dysfunctional phenotypes of growth and Mn accumulation were also demonstrated in mature rice grown in field trials under natural conditions. These results indicate that OsTMN11 plays a primary role in Mn homeostasis and detoxification in rice. To date, no report is available on the functional role of TMN family genes in rice plants.

OsTMN11 was ubiquitously expressed throughout the lifespan of rice and transcripts were abundantly accumulated in the roots and seeds. Deprivation of Mn in the nutrient solution hardly affected OsTMN11 expression in rice at the early vegetative stage. In contrast, overloading Mn in young rice drastically induced the expression of OsMTP11. The potential function related to OsMTP11 expression may be critical for juvenile rice growing under excess Mn stress, since rice crops at this stage in actual farmland usually experience a long-term semi-submerged process in which Mn ions are abundantly absorbed. Our short-term hydroponic study showed that disruption of OsMTP11 compromised the growth of RNAi plants, which displayed repressed plant height, root elongation, and dry biomass under an oversupply of Mn. Analysis of chlorophyll concentrations revealed that the RNAi plants contained significantly lower levels than wild-type rice. The reduced chlorophyll levels should be associated with Mn accumulation in rice because Mn concentrations in both root and shoot tissues were abnormally increased in the RNAi plants. These results suggested that disruption of OsTMN11 could impair rice growth, likely due to overaccumulation of Mn in plants.

The Golgi apparatus is believed to be the center of the protein processing, trafficking, and degradation pathways [29]. Metal (e.g., Ca, Mn or Zn) homeostasis is necessary for the maintenance of the basic Golgi structure and glycosylation activity in organisms [30,31]. Disturbance of Mn homeostasis in the Golgi apparatus of mammalian cells results in abnormal protein processing, which is associated with diseases such as a psychomotor disability, microcephaly, facial hypoplasia, hypotonia, seizures, cardiac defects, and hepatosplenomegaly with increased serum transaminases [32]. Mutation of Golgi Mn transporters TM9SF4 (a V-type ATPase-associated protein belonging to the Golgi-resident TMN family member) and TMEM165 (Ca/Mn pump, a calcium-permeable channel), which are responsible for transport of Mn or other metals in or out of the Golgi, also cause dysfunctions in cell division and differentiation in mammalian cells that evoke pathogenesis and clinical diseases [17,30,32,33]. The importance of Golgi Mn homeostasis has also been demonstrated by several non-TM9 family transport proteins, such as AtMTP11 [25], OsMTP11 [34,35,36], PML3 (UPF0016) [37], BnMTP9 [38], and BICAT3 (BIVALENT CATION TRANSPORTER 3) [39], and disruption of these transport proteins can lead to impaired growth and development by blocking vesicle secretory pathways or cell wall polysaccharide biosynthesis under elevated or normal Mn supply conditions. These results strongly suggest that OsTMN11-mediated Mn homeostasis is also necessary to maintain the processing of growth-related proteins in the Golgi. Similarly, under field conditions (normal Mn supply), the impairment of Golgi Mn homeostasis caused by suppressed OsTMN11 expression might be connected to the phenotype of lower Mn accumulation and reduced reproductivity of the mature RNAi plants. Here, an interesting question may be raised about why a subset of Mn transport proteins encoded by a different gene family is embedded in the Golgi apparatus? Are Golgi Mn transporters from a different family associated with specific processing of Golgi proteins and consequently give rise to different growth and physiological phenotypes? Is there any crosstalk between them? These fundamental and interesting questions are yet to be addressed.

Ectopic OsTMN11 expression in a Golgi-localized Mn exporter *pmrl* mutant of yeast [23] revealed that OsTMN11 can complement the defective phenotype, accompanied by a low level of Mn accumulation compared to the empty vector cells under excess Mn conditions. This observation may allow us to infer that OsTMN11 detoxified Mn ions through a mechanism similar to PMR1 in yeast. PMR1 is believed to mediate Mn homeostasis by loading excessive Mn(II) ions into secretory vesicles to get them out of the cells (exocytosis) [36]. The subcellular phenotype of OsTMN11-mediated Mn efflux is consistent with the observation in yeast, which may also explain why OsTMN11 knockdown led to greater Mn accumulation and supersensitivity to Mn (Figure 4). The suggested working model of OsTMN11 in regulating Mn homeostasis can be further reinforced by the Golgi-localized Mn transporter AtMTP11, the expression of which in *pmr1* yeast restored Mn tolerance [25]. Importantly, while the *atmtp11* mutant was supersensitive to enhanced levels of Mn, detectable microsomes in yeast expressing AtMTP11 showed Mn absorption, suggesting that AtMTP11 mediates Mn homeostasis through a secretory mechanism [34]. Together, these results imply that OsTMN11 may have a mechanism similar to that of AtMTP11 for Mn detoxification through the exocytosis pathway to create Mn efflux. However, whether these Golgi-localized Mn transporters share a similar resistance mechanism for Mn detoxification and how they coordinate Mn homeostatic regulation under Mn stress remains to be investigated.

To validate the phenotypes of young rice regulated by OsTMN11, we additionally conducted a field trial with RNAi and wild-type plants grown under natural conditions (normal Mn supply) until seed maturity. OsTMN11 knockdown also compromised the growth and development, as evidenced by reduced plant elongation, panicle length, seed size, panicle number, spikelet fertility, and seed production. However, the RNAi plants accumulated less Mn in their leaves and rice grains. This indicated that even under normal Mn supply conditions, the reduced expression of OsTMN11 can also exert a long-standing impact on Mn accumulation and homeostasis. The underlying mechanism is not fully understood, but it might be associated with OsTMN11 as an efflux metal transporter, as knockdown of OsTMN11 can retain more Mn in the root cells, which was consistent with the predominant expression of OsTMN11 in the root tissue (Figure 2). If this is the case, the reduced Mn accumulation in rice could be connected to impaired Mn-relevant biochemical and physiological processes, including photosynthesis and consequent seed development [2,22].

Different from Mn, Cd is a non-essential and poisonous metal in the environment [40]. Because Cd and Mn share some similar chemical properties, they are substituted on many metalloproteins, such as chaperones and transporters [2,6]. The present experiments showed that Cd exposure significantly upregulated OsTMN11 expression in rice. For this reason, we took an advantage of a Cd-sensitive *ycf1* mutant strain (YCF1 is a tonoplast Cd influx transporter) that expressed OsTMN11 to identify Cd transport capability. The *OsTMN11*-transformed *ycf1* cells displayed a similar Cd-sensitive phenotype but contained less Cd than the untransformed cells, confirming that OsTMN11 is capable of exporting Cd.

Likewise, a parallel short-term hydroponic study revealed that exposure to low and high levels of Cd led to RNAi plants being more vulnerable to Cd toxicity. Furthermore, the long-term field trial showed that the growth and development of the RNAi lines were also adversely impacted, further supporting the requirement of OsTMN11 for Cd resistance. The weaker growth fitness to Cd stress could be also linked to the Cd accumulation in the RNAi plants. In this case, OsTMN11 may use a similar mechanism for transporting Cd. However, the mature RNAi plants accumulated more Cd in their basal leaves, internodes, and grains, but less Cd in their upper leaves compared to the wild-type rice. The mechanism underlying the phenotype of Cd accumulation would be associated with high expression of OsTMN11 in the roots, with disruption of OsTMN11 causing more Cd accumulation in the roots and basal region of shoots.

## 4. Conclusions

This study functionally identified a novel Golgi-resident metal transporter, TMN11, which belongs to the transmembrane nine protein family, in rice plants. OsTMN11 was demonstrated to be actively involved in Mn export from cells. Knocking down OsTMN11 resulted in Mn overaccumulation in rice seedlings and sensitive growth phenotypes under excess Mn supply conditions, suggesting that OsTMN11 may play a primary role in Mn efflux or homeostasis. The dysfunction in the RNAi plants was also connected to compromised rice growth and seed development with lower Mn accumulation under natural conditions. This is the first instance of functional identification of a rice TM9 family member, which will advance our understanding of the regulatory mechanism of Mn transport, detoxification, and homeostasis in the endomembrane systems associated with rice growth and development. Meanwhile, the RNAi plants may be useful in the phytoremediation of Cd-contaminated wetland soil.

## 5. Materials and Methods

### 5.1. Plant Materials and Treatment

Rice seeds (*Oryza sativa* L. cv. Nipponbare) were surface-sterilized and placed in an incubation chamber at 28 ± 2 °C under darkness for 3 days. After germination, the seedlings were transferred to a clean mesh sieve floating on 1/2 Kimura B nutrient solution (pH 5.6) and grown under 250 μmol m^−2^ s^−1^ photon density at 30 °C for 16 h light/25 °C for 8 h dark. For metal treatment, two week-old rice plants were grown in renewed nutrient solutions with or without Mn (MnCl_2_) or Cd (CdCl_2_). The treatment concentrations of Mn, Cd, Zn (ZnSO_4_), and Fe (FeSO_4_) depended on the experiments required. After treatment, the shoots and roots were harvested and the fresh tissues were used for growth and metal determination.

The field trials for lifelong rice growth and development were separately conducted at the Research Station of NAU, where two types of local paddy soils were used for the studies. The farmland for the Mn absorption experiment contained yellow brown soil (YBS) with the physical and chemical properties described previously [22], and the Cd-contaminated paddy soil was brown sand clay (BSC) with 0.41 mg·kg^−1^ total Cd [41]. Three week-old rice plantlets growing in the hydroponic medium were transplanted in the open paddy soils. For each trial of the study, the area of the field was divided into four plots and each plot was arranged with at least three replicates. Prevalent agronomic management practices were adopted and the rice grew naturally to maturity from 28 May to 30 September 2021. Local meteorological data recorded no abnormal rainfall, temperature, pest, or disease occurrences, etc. When the rice was totally ripened, all of the tissues and organs of the mature plants were separately harvested [7].

### 5.2. Analysis of Bioinformatics Related to OsTMN11 Gene and Protein Sequences

The coding region sequence of *OsTMN11* was cloned and sequenced. The isolated sequence was mapped to the database from the rice genome (http://rice.plantbiology.msu.edu, accessed on 20 November 2022) according to the ID name OsTMN11 (LOC_Os02g554400). The potential domains of the OsTMN11 protein were predicted using SMART (Simple Modular Architecture Research Tool) (http://smart.embl-heidelberg.de/, accessed on 20 November 2022). The HMM model data of the EMP70 domain was obtained from Pfam database (http://pfam.xfam.org, accessed on 20 November 2022) and proteins with the same domains in Arabidopsis and rice were screened using HMMER3.0 software. Sequence alignment and phylogenetic tree analysis of the selected genes were carried out using MEGA7.0 software.

### 5.3. Transcriptional Expression of OsTMN11

Analysis of *OsTMN11* transcripts was performed based on a previously described method [42]. Briefly, the rice plantlets were hydroponically grown until maturity. The roots, shoots, and other tissues or organs were separately sampled. Total RNA was extracted using Trizol reagent (Invitrogen, Shanghai, China) and specifically designed primers (Table S1). DNA was removed using a one-step method, while cDNA was synthesized using reverse transcriptase SuperMix (Transgene, Beijing, China). The cDNA of *OsTMN11* was amplified using iTaq^TM^ Universal SYBR Green Supermix (Bio-Rad, Hercules, CA, USA). The purity and concentration of the RNA were detected using a Nano-400 ultraviolet microspectrophotometer. The qRT-PCR reactions were carried out using SYBR^®^ Green premix Ex Taq^TM^ (TaKaRa, Shanghai, China) reagent. The reaction was performed using the 7500 real-time PCR System (Applied Biosystems, Foster City, CA, USA). The samples were pre-incubated at 94 °C for 30 s, followed by 45 cycles of denaturation at 94 °C for 10 s and annealing at 60 °C for 30 s.

### 5.4. Generation of Rice RNAi Lines

The specific *OsTMN11* sequence with a 365-bp fragment was selected and amplified to generate the RNAi construct using the specific forward and reverse primers (Table S1). The fragment was subcloned into the KpnI/SacI sites of the LH-FAD2-1390 RNAi vector and the inverse DNA fragment was also amplified into BamHI/PstI sites of the same vector [43]. The forward fragment was first connected to the RNAi-1390 vector, and then the reverse fragment was connected to the RNAi-1390+ forward vector. The fragments were transformed into *Escherichia coli* to gain the 1390-OsPDR20-RNAi plasmid. The embryogenic callus of rice induced by mature embryo was infected. At least twenty RNAi lines (T3 homozygotes) were obtained. Three RNAi lines (RNAi-3, RNAi-7, and RNAi-9) were randomly selected for the functional study.

### 5.5. Analysis of Subcellular Localization of OsTMN11

The OsTMN11 sequence was isolated and amplified by RT-PCR using the specific premiers described above (Table S1). The corresponding N-terminal or C-terminal sequences were separately connected with green fluorescent protein (GFP) at the Bgl2/Spe1 site by the restriction endonuclease, and each was inserted into the pCambia1300-GFP containing (35S-GFP) vector driven by the 35S promoter. The pCambia1300-GFP vector without TMN11 was used as a control. The 35S-OsTMN11-GFP and specific markers (including *cis*-Golgi Man-1-mRFP, TGN mRFP-SYP61, plasma membrane PIP2A, nuclear OsMADS3, and endoplasmic reticulum KDEL) were co-expressed in the tobacco leaf epidermal cells mediated by *Agrobacterium tumefaciens* GV3101 strain, incubated for 2–3 days, and visualized using confocal laser scanning microscopy (Confocal System-UitraView VOX, Perkin Elmer) [22].

### 5.6. Transformation OsTMN11 into Yeast Cells

The *OsTMN11* sequence was amplified using primers (Table S1) and transformed into the wild-type BY4741 yeast strain (*Saccharomyces cerevisiae*) and metal-sensitive mutants *pmr1* (Mn), *ycf1* (Cd), *zrc1* (Zn), and *ccc1* (Fe) lacking transport activities (BY4741 background) [44]. The empty pYES2 vector was used as a control. Two expression vectors (pYES2-*OsTMN11* and pYES2) were separately transformed into the metal-sensitive mutants and the wide-type strain. The gene-positive yeast clones were incubated until the concentration ranged within an OD600 value of 0.8–1.0. The cell dot plate experiment was carried out on synthetic galactose uracil solid (YNB) medium containing 0, 2, 4, and 8 µM for Mn and Zn; 0, 5, 30, and 60 µM for Cd; and 0, 6, 8, and 10 µM for Fe. The content gradient 100, 10^−1^, 10^−2^, and 10^−3^ was used for YNB liquid medium dilution and cells were incubated for 2 days. The corresponding mutants connected with pYES2 and *OsTMN11* were cultured in the liquid YNB medium with respective metals at 28 °C for 48 h, and then the concentrations of metals were measured.

### 5.7. Measurement of Metal Concentration

The yeast cells and rice tissues and organs, such as roots, leaves, stem, husk, brown rice, etc., were separately harvested, washed three times with 5 mM cooled EDTA (4 °C, pH 5.0), and dried at 75 °C for 24–60 h. The dry weight of samples was recorded and digested in a solution of pure nitric acid in a microwave digestion/extraction system at 120 °C for 20–60 min. The metal concentration was quantified using inductively coupled plasma optical emission spectrometry (ICP-OES; Optima 2100DV, Perkin Elmer Instruments, Waltham, MA, USA) [45].

### 5.8. Measurement of Chlorophyll and Malondialdehyde

Rice leaves were harvested, washed, and submerged in 80% acetone overnight until the leaves were bleached. The absorbance of the chlorophyll extract was measured using a spectrophotometer at 663/645 nm [46].

### 5.9. Analysis of Morphology and Phenotypes Relevant to Seed Development

Survey and analysis of mature rice structures and grain yields were conducted using previously described methods [7]. Briefly, the ripening rice plants were collected from the field, and their tissues and organs were separately sampled and surveyed in the lab. Most of the growth and development indexes were carefully analyzed, such as shoot height, internode length, panicle, seed weight per plant, morphology of seed width and length, spikelet fertility per panicle, 1000-grain weight, and grain yield per square meter.

### 5.10. Statistical Analysis

For each treatment, 16–20 rice plants were set up with biological triplicates. IBM SPSS 20.0 software (IBM Crop., Armonk, NY, USA) was used for statistical analysis. Analysis of variance (ANOVA) was applied for evaluating significant differences between treatment and control groups. The data were calculated as the mean ± standard deviation of triplicate. Significant differences between treatments were statistically evaluated by standard deviation and analysis of variance (Tukey’s test, *p* < 0.05).

## Data Availability

Not applicable.

## Supplementary Materials

The following supporting information can be downloaded at: https://www.mdpi.com/article/10.3390/ijms232415883/s1, Figure S1: Diagram of localization of OsTMN11 in rice chromosome 2; Figure S2: Transcriptional expression patterns of OsTMN11 under normal (control) and excess Zn and Fe; Figure S3: Analysis of transcripts of OsTMN11 knockdown mutants by RNA interference (RNAi); Figure S4: Growth response of WT and RNAi lines under excessive Mn and Cd stress; Figure S5: Growth response of wild-type (WT) and RNAi plants under Mn deficiency; Figure S6: Morphological differences in tissues and organs related to seed development between wild-type (WT) and RNAi rice plants grown in Cd-contaminated field soil; Figure S7: Seed morphological differences between wild-type (WT) and RNAi rice plants grown in Cd-contaminated field soil; Table S1: Primer sequences used for this study.
