# Supplementary material for: A Golgi-Located Transmembrane Nine Protein Gene TMN11 Functions in Manganese/Cadmium Homeostasis and Regulates Growth and Seed Development in Rice
Source: Int J Mol Sci. 2022 Dec 14;23(24):15883. doi: 10.3390/ijms232415883 (PMC9779671; doi:10.3390/ijms232415883)
Supplement: Supplementary file 1 [file ijms-23-15883-s001.zip › ijms-2089104-supplementary.pdf]

*Supplementary*

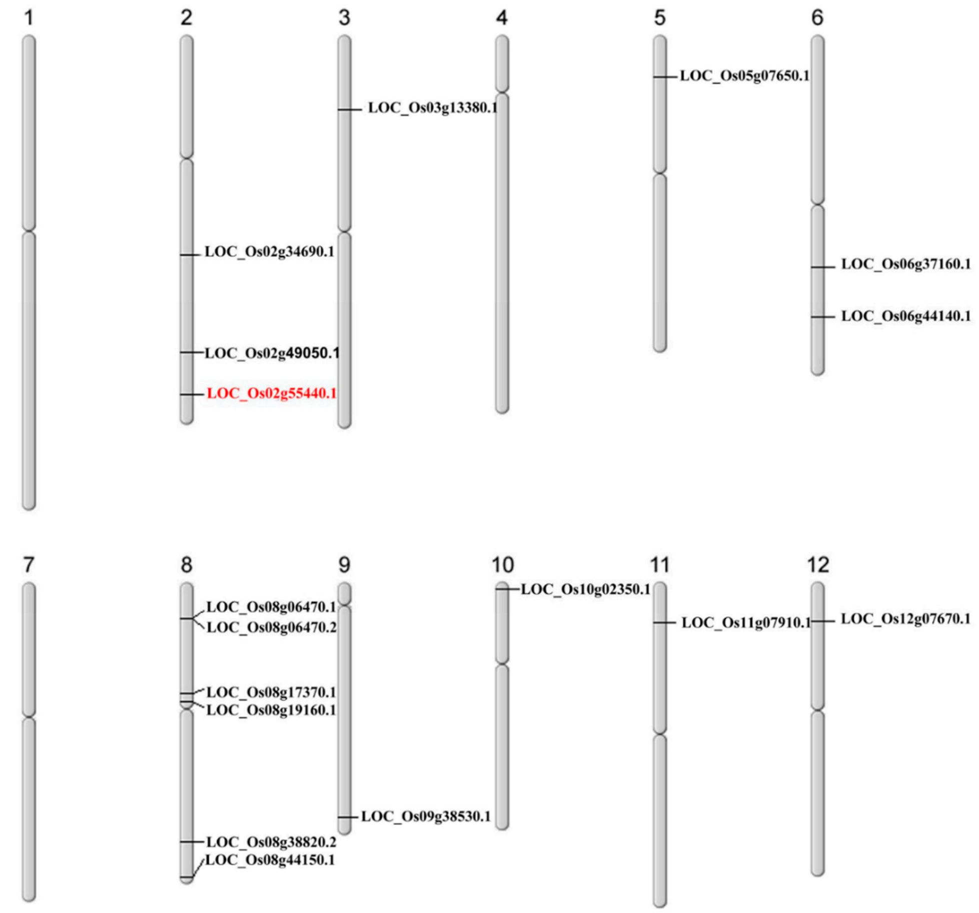


**Figure S1.** Diagram of localization of *OsTMN11* in rice chromosome 2.

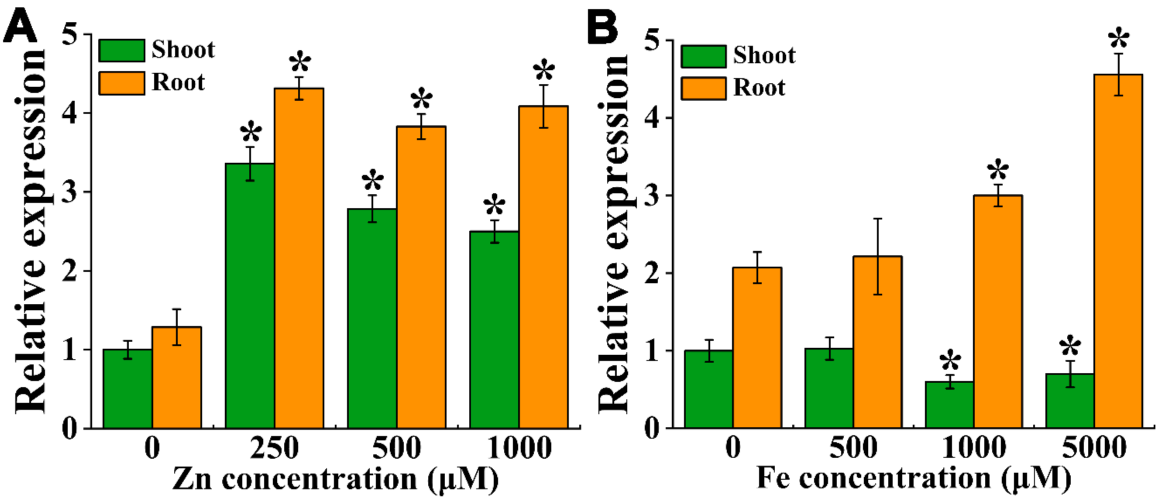


**Figure S2.** Transcriptional expression patterns of *OsTMN11* under normal (control) and excess Zn and Fe. Two weeks-old rice seedlings were exposed to 0, 250, 500, 1000 μM Zn (A) or 0, 500, 1000, 5000 μM Fe (B) for 6 hours. *OsTMN11* transcripts were measured by PCR. Bars indicate means ± standard deviations of three biological replicates. Asterisks indicate that the mean values are significantly different between the treatment and control ($p$<0.05).

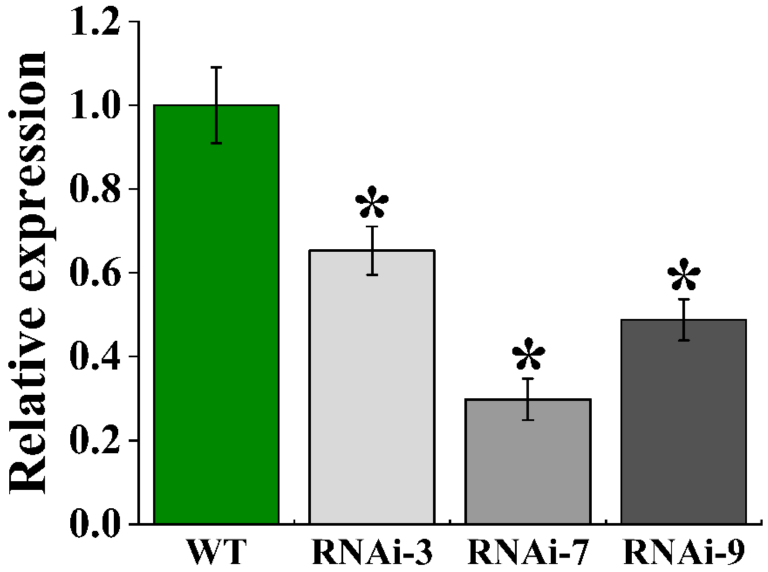


**Figure S3.** Analysis of transcripts of *OsTMN11* knockdown mutants by RNA interference (RNAi). The transcript levels of *OsTMN11* in WT and RNAi strains (RNAi-3, RNAi-7, and RNAi-9). Bars indicate means ± standard deviations of three biological replicates. Asterisks indicate that the mean values of three replicates are significantly different between WT and RNAi lines ($p$<0.05).

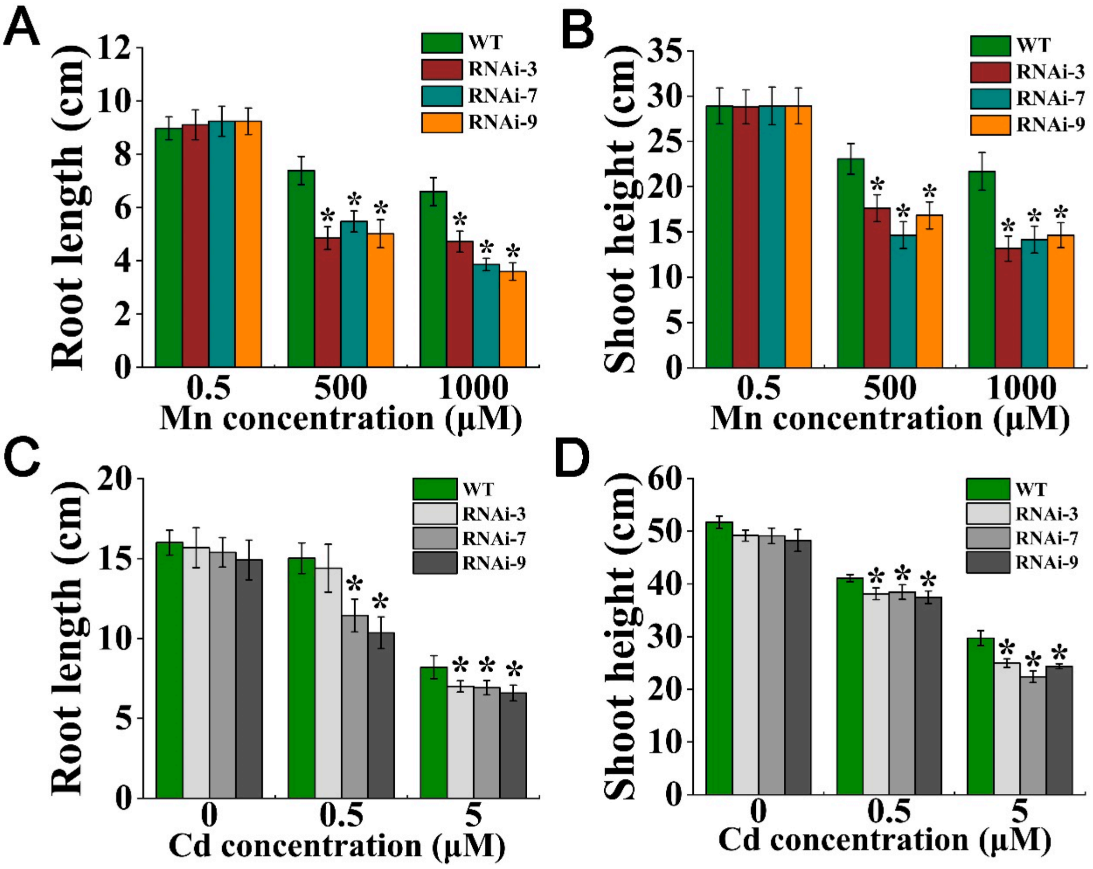


**Figure S4.** Growth responses of WT and RNAi lines to excessive Mn and Cd stress. Two weeks-old young rice plants were grown in the nutrient solution supplemented with 0.5 (normal), 500, and 1000 μM Mn or 0, 0.5, and 5 μM Cd for 14 days. (A), (B) Root and shoot elongation under Mn stress. (C), (D) Root and shoot elongation under Cd stress. Bars indicate means ± standard deviations of three biological replicates. Asterisks indicate that the mean values of three replicates are significantly different between WT and RNAi lines ($p<0.05$).

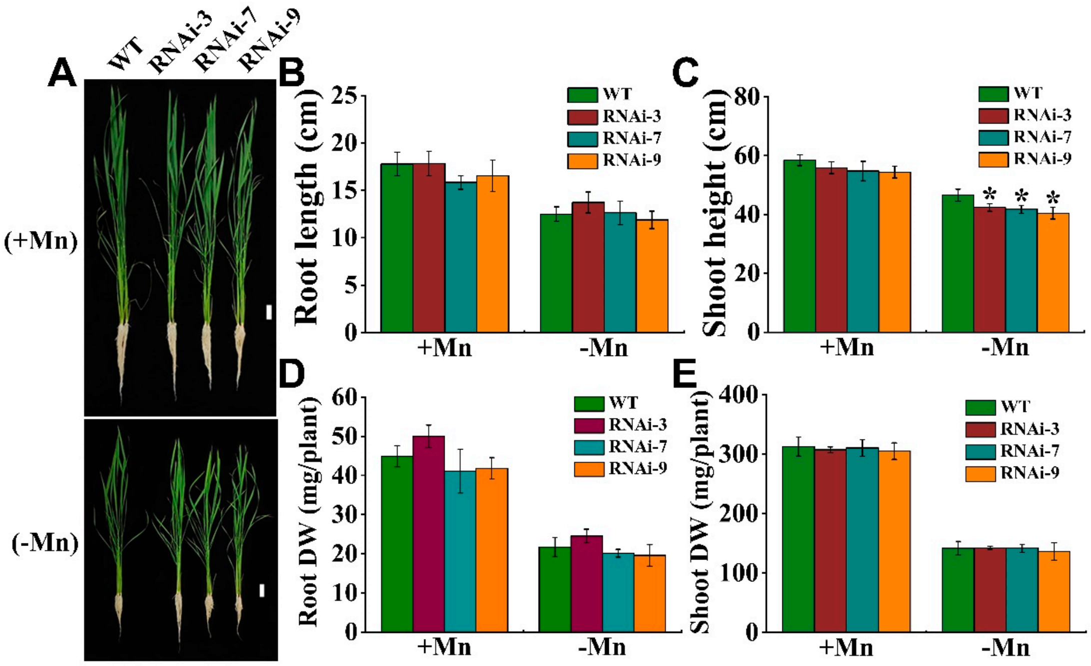


**Figure S5.** Growth response of wild-type (WT) and RNAi plants under Mn deficiency. Two weeks-old rice was cultured in Mn-deficient and normal nutrient solution for 30 d. (A) Phenotype of WT and RNAi lines under Mn deficiency. Bars = 3 cm. (B) Root length. (C) Shoot height. (D) Root dry weight (DW). (E) Shoot DW. Bars indicate means ± standard deviations of three biological replicates. Asterisks indicate that the mean values of three replicates are significantly different between WT and RNAi lines ($p<0.05$).

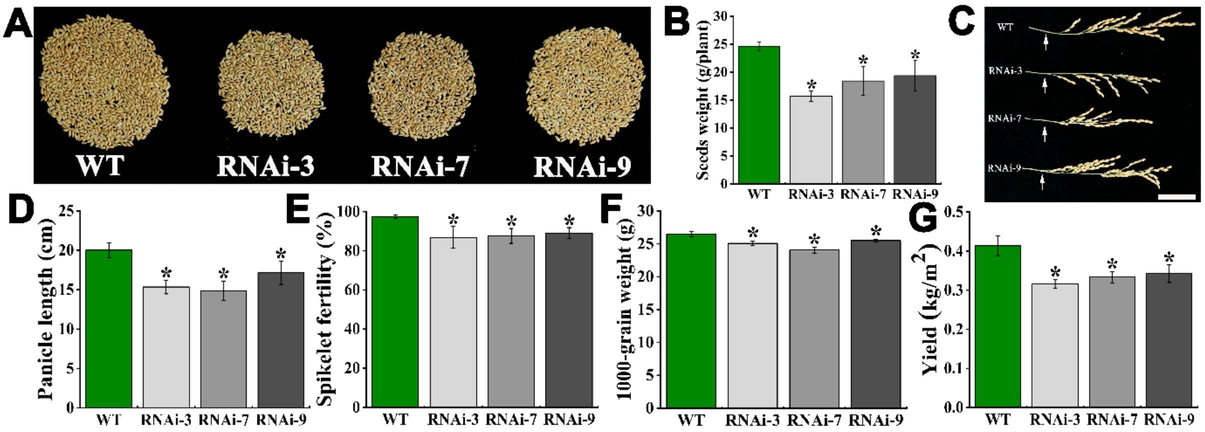


**Figure 6S.** Morphological differences of tissues and organs related to seed development between wild-type (WT) and RNAi rice plants grown in the Cd-contaminated field soil. The rice varieties grew up to ripening under natural conditions. (A), (B) Seed weight per plant. (C) Phenotypes of panicles (Bars = 5 cm). (D) Panicle length. (E) Spikelet fertility of a panicle. (F) 1000-grain weight. (G) Seed yield per square meter. Bars indicate means ± standard deviations of at least three independent biological replicates. Asterisks indicate that the mean values of three replicates are significantly different between WT and RNAi lines ($p$<0.05).

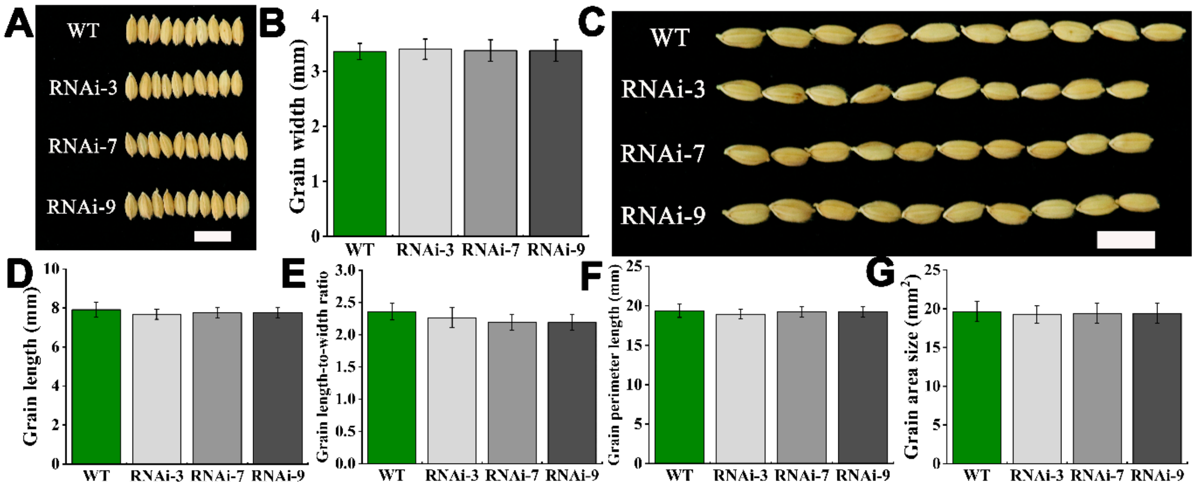


**Figure S7.** Seed morphological differences between wild-type (WT) and RNAi rice plants grown in the Cd-contaminated field soil. The rice varieties grew up to ripening under natural conditions. (A), (B) Grain width. (C), (D) Grain length. Grain length-to-width (E), perimeter length (F) and area size (G). Bar = 5 cm (A and C). Bars indicate means ± standard deviations of at least three independent biological replicates.

**Table S1.** Primer sequences used for this study

| Primer name | Primer sequence（5' to 3'） | Function |
|---|---|---|
| OsTMN11-F | ATGGCGCGCCTCCTCCCCTT | OsTMN11 gene full-length cloning |
| OsTMN11-R | TTAATCTGCTTTCACGGATG | |
| OsTMN11-GFP-F | GGATCCAGATCAAAACCACACGC | OsTMN11-GFP fusion vector |
| OsTMN11-GFP-R | ATCTGCTTTCACGGATGAG | |
| RNAi-F1 | GGGGTACCCCTCTATCTCGGCTACTCACTC | *RNA i*nterference vector |
| RNAi-R1 | CGAGCTCGGCAGCAGAAAGAGCAACTAA | |
| RNAi-F2 | CGGGATCCCCTCTATCTCGGCTACTCACTC | |
| RNAi-R2 | AACTGCAGGGCAGCAGAAAGAGCAACTAA | |
| qRT-PCR-F | GCATTCCTGATCCTTACCACTC | qRT-PCR analysis |
| qRT-PCR-R | AAGCGGCACAGAGATACAGA | |
| *OsActin*-F | GAGTATGATGAGTCGGGTCCAG | |
| *OsActin*-R | ACACCAACAATCCCAAACAGAG | |
| *OsUbiqutin-F* | CGCAAGTACAACCAGGACAA | |
| *OsUbiqutin-R* | TGGTTGCTGTGACCACACTT | |
| pYES2-F | AATATACCTCTATACTTTAACGTC | Yeast expression vector |
| pYES2-R | GCGTGAATGTAAGCGTGAC | |
